# Substitution of Carbohydrates for Fats and Risk of Type 2 Diabetes among Korean Middle-Aged Adults: Findings from the Korean Genome and Epidemiology Study

**DOI:** 10.3390/nu14030654

**Published:** 2022-02-03

**Authors:** Hye-Ah Lee, Hyesook Park

**Affiliations:** 1 Clinical Trial Center, Ewha Womans University Mokdong Hospital, Seoul 07985, Korea; 2 Department of Preventive Medicine, College of Medicine, Ewha Womans University, Seoul 07804, Korea; hpark@ewha.ac.kr; 3 Department of Preventive Medicine, Graduate Program in System Health Science and Engineering, Ewha Womans University, Seoul 07804, Korea

**Keywords:** cohort study, diabetes, interaction, macronutrient

## Abstract

Using data from a 16 year follow-up cohort of the Korean Genome Epidemiology Study, this study assessed the effects of carbohydrate intake on incident diabetes, including replacement of fats or proteins with carbohydrates. In addition, this study evaluated modification effects based on 24 genetic variants associated with type 2 diabetes. For the daily intake of macronutrients, the energy-adjusted intake and percentage of total energy intake were calculated. The effects were assessed using a Cox proportional hazards model; results were presented as hazard ratios with 95% confidence intervals (CIs). Among the 7413 participants considered to be diabetes-free at baseline, 1193 individuals were considered to have incident diabetes. The risk of incident diabetes was found to be high at both extremes of carbohydrate intake, with the lowest risk at 78 E%. The replacement of 5 E% intake from fats with isocaloric carbohydrates showed an 11% increase in the risk of diabetes (95% CI: 1.01–1.21), which was significant in men, participants >50 years of age, and participants with a high educational level. Regarding gene–environment interactions, the relationship between carbohydrate intake and incident diabetes was not dependent on genetic variants. A nonlinear relationship was observed between carbohydrate intake and incident diabetes. The substitution of carbohydrates for fats was also associated with an increased risk of incident diabetes.

## 1. Introduction

Based on a recent global burden-of-disease study, 476.0 million individuals had diabetes in 2017, an increase of 129.7% from 211.2 million in 1990 [1]. Furthermore, in the Republic of Korea, the prevalence of diabetes increased from 8.6% in 2001 to 13.8% in 2018 among adults >30 years of age [2]. According to the findings from the 2012 Korean burden-of-disease study, diabetes constituted the primary cause of disease burden [3]. Hence, the primary prevention of diabetes is a major public-health concern.

Diet is a major factor for diabetes management and prevention. Among dietary macronutrients, carbohydrates directly affect insulin secretion and blood sugar [4]. Regarding the effect of carbohydrate intake on health risk, associations with hypo-HDL cholesterolemia [5], mortality [6], and type 2 diabetes [7,8] have been reported. However, because of inconsistent results in previous studies, there remain questions regarding the effects of carbohydrate intake on diabetes. In a systematic review that included randomized controlled trials with a duration of at least 6 months, low-carbohydrate diets reportedly helped to improve glycemic control but not weight loss [9]. Meanwhile, European studies with long-term follow-up periods have shown an inverse association between carbohydrate intake and the risk of type 2 diabetes [10,11]. Other studies have shown a null association [12,13]. To evaluate the effects of diet, it is necessary to study the effects in various countries, considering cultural, social, and environmental differences. Macronutrient intake was also evaluated in terms of substitution in a previous study. Furthermore, several studies have evaluated gene–environment interactions to provide evidence for precision medicine [14,15]. Although gene–environment interactions were reported in several studies [16,17], there remains insufficient evidence. Moreover, there is limited knowledge regarding gene–macronutrient interactions, due to the need to control for adequate confounding variables and consider the effect of isocaloric macronutrient substitution [14].

Thus, using 16-year follow-up data from a study of middle-aged Koreans, this study evaluated the association between incident diabetes and carbohydrate intake, as well as the substitution of carbohydrates for fats or proteins. Genetic variants associated with type 2 diabetes were reported in a recent study [18]. Therefore, for these variants, the interaction between genetic variants and carbohydrate intake was evaluated in this study.

## 2. Methods

### 2.1. Study Participants

This study used data from the community-based cohort in the Korean Genome and Epidemiology Study (KoGES). KoGES consists of six prospective cohort studies, and the community-based cohort has the longest follow-up period. Detailed information concerning this cohort has been published elsewhere [19]. In brief, to investigate risk factors for chronic diseases in Koreans, the community-based cohort of the KoGES was implemented in 2001–2002; a follow-up survey is conducted biennially. The study participants consisted of 10,030 volunteers between 40 and 69 years of age who lived in Ansung (*n* = 5018, a rural region) and Ansan (*n* = 5012, an industrial region), both in Gyeonggi Province. The study participants completed the baseline survey. The follow-up survey included data associated with demographic factors, disease history, and health-related behaviors; data were collected via questionnaires, anthropometric measurements, and biomarker assays. Because it takes time to update the database and release data, survey data up to the 8th follow-up are now available. Thus, the present study includes data up to the eighth follow-up (conducted in 2017–2018; follow-up rate = 61.4%).

In this study, individuals who met any of the following criteria were excluded: history of cancer (any type), myocardial infarction, stroke, coronary artery disease, or congestive heart failure (*n* = 1274); history of diabetes, fasting glucose ≥126 mg/dL at baseline, or hemoglobin A1c (HbA1c) ≥ 6.5% at baseline (*n* = 1029); missing dietary survey data at baseline (*n* = 248); and daily caloric intake of <500 kcal or >5000 kcal (*n* = 66). Finally, data for 7413 participants (3507 men and 3906 women) were analyzed. In addition, when evaluating interactions with genetic variants, 6017 participants were analyzed (excluding individuals with missing genetic data). Compared with the participants in this study, the excluded individuals were slightly older and had a higher body mass index (BMI) and a lower physical-activity level; however, their sex distribution and macronutrient intake (E%) did not differ (data not shown). The study protocol was approved by the Institutional Review Board (IRB) of Ewha Womans University Hospital (IRB no. EUMC 2021–03–008).

### 2.2. Diabetes Ascertainment

During the 16 year follow-up period, incident diabetes was identified based on the following criteria: self-reported physician-diagnosed diabetes, a fasting glucose concentration ≥126 mg/dL, or HbA1c ≥6.5%. Follow-up began upon entry into the study and ended on the date of physician-diagnosed diabetes, detection of a fasting glucose concentration ≥126 mg/dL, or detection of HbA1c ≥6.5% during the follow-up period, or at the end of the follow-up period (whichever was earlier).

### 2.3. Macronutrient Intake

At baseline, mean food intake over the past year was investigated using a dish-based semi-quantitative food-frequency questionnaire (FFQ) with acceptable validity and reliability (consisting of 103 food items) [20]. Based on the frequency of consumption and the portion size of each food item, the daily intake of nutrients was calculated. The KoGES provides data concerning the intake of 24 nutrients, including total energy. Using data regarding the daily intake of macronutrients, the energy-adjusted intake of macronutrients was estimated using the residuals method. The macronutrient intake was also calculated as a percentage of total energy intake (E%) and divided into quintiles (<65.6 E%, 65.6–69.7 E%, 69.7–73.3 E%, 73.3–77.0 E%, and ≥77.0 E%).

### 2.4. Covariates

Based on the findings in previous studies [13,16], the following covariates were considered: sex, age, rural region, educational level (did not graduate high school/graduated high school/at least some college), current smoking status, alcohol intake, physical activity, and BMI at baseline. Alcohol intake was measured in grams per day and categorized according to the criteria in a previous study (no alcohol, <15 g/day, 15–24.9 g/day, and ≥25 g/day) [21]. Physical activity over the past year was evaluated using the International Physical Activity Questionnaire [22] to measure the metabolic-equivalent task hours per week and then categorized into quartiles.

The community-based cohort data include exome chip data. In a recent study by Cho et al. [18], 23 variants were reportedly associated with type 2 diabetes based on meta-analysis using exome chip KoGES data and the GWAS Catalog database, together with six new variants related to type 2 diabetes. Among the variants, rs10440833, rs4712523, rs7754840, rs4712524, rs10946398, and rs9295474 in *CDKAL1* showed strong linkages (*r*^2^ ≥ 0.8) in linkage disequilibrium analysis. Therefore, only rs7754840 of *CDKAL1* was considered in the present study; the interaction effects for 24 variants were assessed using a multiplicative scale.

### 2.5. Statistical Analysis

Participant characteristics are shown as means with standard deviations for continuous data and frequencies with percentages for categorical data. Differences in participant characteristics according to quintile of carbohydrate intake (E%) were assessed using analysis of variance or the chi-squared test.

The incidence rate of diabetes was estimated per 1000 person-years. The risk of incident diabetes was evaluated using a Cox proportional hazards model; thus, results are shown as hazard ratios (HRs) with 95% confidence intervals (95% CIs). Proportional hazards assumptions were satisfied on the basis of Schoenfeld residual and log-minus-log plot results. The risk of diabetes according to quintile of carbohydrate intake (E%) was evaluated. The upper limit of carbohydrates from the acceptable macronutrient distribution ranges suggested by the Dietary Reference Intake for Koreans 2015 is 65 E% [23]. Therefore, the risk of incident diabetes was assessed based on ≥65 E% carbohydrate intake. In multivariate analysis, HRs with 95% CIs were estimated after adjustments for sex, age, region of residence, educational level, current smoking status, alcohol intake, quartiles of physical activity, and BMI at baseline. To evaluate the carbohydrate intake associated with a low risk of diabetes, carbohydrates were evaluated as a continuous variable in a restricted cubic spline regression model.

To evaluate the association between incident diabetes and substitution of carbohydrates for fats or proteins, multivariate nutrient-density models (energy-yielding nutrients as E%) and nutrient-residual models (energy-adjusted nutrients as g/day) were used [24]. Thus, carbohydrate, protein, and total energy were included as covariates for the substitution of carbohydrates for fats; carbohydrates, fats, and total energy were included as covariates for the substitution of carbohydrates for proteins. To assess nonlinearity, restricted cubic spline regression was used. Based on a low Akaike information criterion value for model fit, four knots were selected. The reference value was fixed at 65 E% of carbohydrate.

In addition, to evaluate interaction effects with genetic variants, genetic variants were included as an additive model; interaction effects were evaluated based on a multiplicative scale using the Wald test. To avoid false positives (i.e., type I errors), the false discovery rate (FDR)-adjusted *p*-value was estimated using the Benjamini–Hochberg method. As a sensitivity analysis, the association after adjusting for waist circumference (WC) instead of BMI was evaluated.

All analyses were performed using SAS version 9.4 (SAS Institute, Cary, NC, USA). Statistical significance was regarded as *p* < 0.05 in two-tailed tests.

## 3. Results

During the 16 year follow-up period, 1193 individuals (13.1/1000 person-years, 95% CI: 12.4–13.9) were considered to have incident diabetes (cumulative incidence: 16.1%). The incidence rate was higher in men (14.0/1000 person-years, 95% CI: 12.9–15.2) than in women (12.3/1000 person-years, 95% CI: 11.4–13.3; *p* = 0.024). At baseline, carbohydrate intake constituted 71.1 E%, fat constituted 14.4 E%, and protein constituted 13.4 E% of the total energy intake. Participants with relatively high carbohydrate intakes were older, had a lower educational level, and tended to be nonsmokers and nondrinkers. The BMI level did not differ according to the quintile of carbohydrate intake, but mean WC showed a significant difference (Table 1).

**Figure 1:**
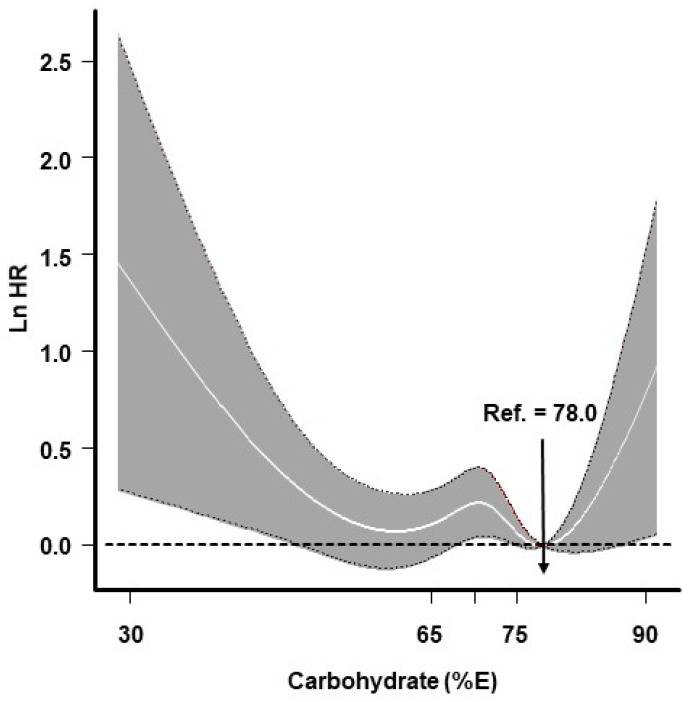
Association between carbohydrate intake (E%) and incident diabetes. Line indicates dose–response curve and gray areas represent 95% confidence interval (CI). Knots were located at the 25th, 50th, and 75th percentiles of carbohydrate-intake distribution. The estimated log hazard ratio (Ln HR) was adjusted for sex, age, rural region, educational level, current smoking status, alcohol intake, physical activity, body mass index (BMI), and total energy. Ref., reference.

The risks of diabetes according to the quintile of carbohydrate intake are shown in Table 2. Participants in the highest carbohydrate quintile had a 1.21-fold (95% CI: 1.01–1.45) greater risk of incident diabetes than did individuals in the lowest carbohydrate quintile; however, this relationship was not statistically significant after adjusting for covariates. When participants were stratified according to sex, the association between carbohydrate intake quintile and incident diabetes showed a dose–response relationship in women but not in men; however, the association did not remain statistically significant after adjustments for covariates. When assessed based on ≥65 E% carbohydrate intake, a null association was observed (data not shown). Furthermore, the effects of carbohydrates on incident diabetes showed a nonlinear relationship with the lowest risk at 78 E%. The risk of incident diabetes was found to be high at both extremes of carbohydrate intake (a U-shaped relationship) (Figure 1).

**Figure 2:**
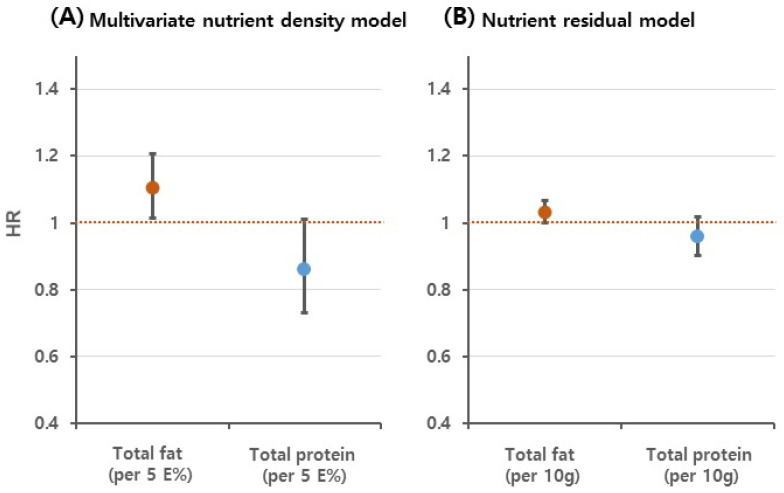
Risk of incident diabetes when carbohydrates were substituted for fats or proteins: (**A**) Multivariate nutrient density model and (**B**) Nutrient residual model. Results are presented as hazard ratio (HR, points) with 95% confidence interval (CI, vertical bars). The red and blue points represent the results of replacement of fats or proteins with carbohydrates, respectively. The multivariate nutrient-density model was adjusted for sex, age, rural region, educational level, current smoking status, alcohol intake, physical activity, body mass index (BMI), total energy, and protein (per 5 E%). In the nutrient-residual model, the energy-adjusted protein (per 10 g) was applied as a covariate.

**Figure 3:**
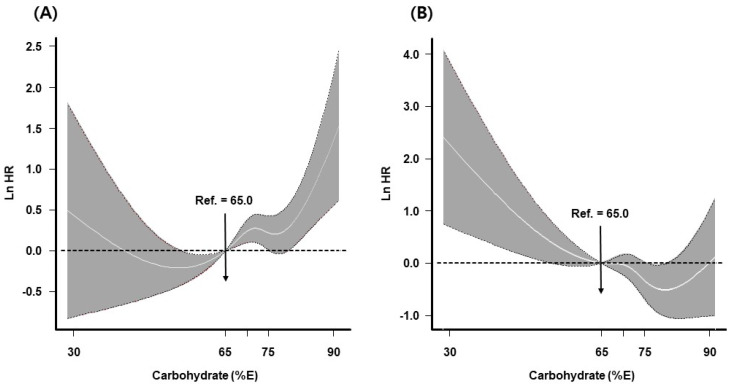
Risk of incident diabetes when carbohydrates were substituted for fats (**A**) or proteins (**B**). Line indicates dose–response curve, and gray areas represent 95% confidence interval (CI). The reference point is 65 E% from carbohydrates (upper limit suggested by the Dietary Reference Intake for Koreans 2015) with knots placed at the 25th, 50th, and 75th percentiles of carbohydrate intake distribution. The estimated log hazard ratio (Ln HR) was adjusted for sex, age, rural region, educational level, current smoking status, alcohol intake, physical activity, body mass index (BMI), total energy, and protein (per 5 E%) when substituting carbohydrates for fats. In the model that evaluated carbohydrate substitution for proteins, fat (per 5 E%) was applied as a covariate. Ref., reference.

Figure 2 and Figure 3, and Table 3 show the results for the replacement of fats or proteins with carbohydrates. Although the substitution of carbohydrates for proteins showed an HR < 1.0, higher carbohydrate intake at the expense of protein was not associated with diabetes risk in either model. Replacement of fats with carbohydrates was significantly associated with diabetes risk (HR for 5 E% substitution 1.11, 95% CI: 1.01–1.21 in the multivariate nutrient-density model; HR for 10 g substitution 1.03, 95% CI: 1.00–1.07 in the nutrient-residual model; Figure 2). When the evaluation was based on stratification according to baseline characteristics, the association was significant in participants ≥50 years of age, men, participants with a high educational level, and participants without abdominal obesity; however, an interaction effect was not observed (Table 3). In the spline model, replacement of proteins with carbohydrates was inversely associated with diabetes risk; however, the risk increased at high carbohydrate levels. Replacement of fats with carbohydrates increased the risk of diabetes at both extremes of carbohydrate intake level (Figure 3).

Regarding modification effects based on genetic variation, the effects of high carbohydrate intake (≥65 E%) differed according to genotype in rs7901695, rs7593730, and rs4430796; these effects were not statistically significant after FDR correction (Supplementary Table S1). In men, the effects of carbohydrate intake >65 E% on the development of diabetes differed according to the rs4430796 genotype in *HNF1B*. However, the FDR-adjusted *p*-value was not statistically significant (Supplementary Table S2). The effects of carbohydrate intake quintiles on incident diabetes did not differ according to genetic variants (data not shown). In addition, significant associations were not observed in either multivariate nutrient-density models or nutrient-residual models when replacing fats with carbohydrates (Supplementary Table S3). In the association analysis, there was no change in the results when WC was adjusted instead of BMI (data not shown).

## 4. Discussion

Using data from a 16 year follow-up cohort, this study assessed the effects of carbohydrate intake on incident diabetes; the analysis included replacement of fats or proteins with carbohydrates, as well as the effects of modification according to genetic variants. The positive association between the quintile of carbohydrate intake and diabetes risk was not statistically significant after adjustments for covariates. When carbohydrate intake was regarded as a continuous variable, carbohydrate intake showed a U-shaped relationship with diabetes. The study results also showed an 11% increase in the risk of diabetes with replacement of 5 E% fat intake with isocaloric carbohydrates; this effect was significant in men, participants >50 years of age, and participants with a high educational level, but no interaction effect was observed. In addition, the substitution effect did not differ according to genetic variants associated with type 2 diabetes.

The results also showed a modification effect of rs4430796 in *HNF1B* on the association between high carbohydrate intake (≥65 E%) and incident diabetes only in men; however, this effect did not remain significant after FDR correction. In a recent study using the same data source as in this study, women with >65 E% carbohydrate intake had a different risk of diabetes based on *AMY1* single-nucleotide polymorphism genotypes (rs6696797, rs4244372, and rs10881197) [16]. In another study that included individuals with type 2 diabetes, the effect of carbohydrate intake (E%) on clinical markers differed according to rs1501299 genotype in *ADIPOQ* [17]. However, small numbers of single nucleotide polymorphisms were evaluated in those two studies. In another study, genetic variants that interact with macronutrient intake for type 2 diabetes risk were systematically reviewed and their effects were evaluated using European Prospective Investigation into Cancer and Nutrition (EPIC) data; however, the study results could not be replicated. That study pointed out that previous studies may have generated false-positive results because they lacked adequate correction for multiple tests [14]. In addition, the differences in dietary evaluation can contribute to discordant findings. The FFQ questionnaire commonly used in observational studies may indicate different amounts of nutrients, depending on the food items listed.

There have been inconsistent conclusions regarding the effects of carbohydrates on diabetes. In a 12-year follow-up study that included 25,943 male smokers, quintiles of carbohydrate intake were associated with a decreased risk of diabetes [10]. In a cross-sectional study in the UK that used National Diet and Nutrition Survey data, each 5 E% decrease in carbohydrate intake was associated with a 12% greater risk of diabetes [11]. In other studies, higher carbohydrate intake was reportedly associated with an increased risk of diabetes [7,25]. In agreement with the present findings, the EPIC-Potsdam Study [12] and the EPIC-Norfolk study [13] showed null associations between carbohydrate intake and incident type 2 diabetes. However, the findings in those studies did not consistently remain significant after adjustments for covariates. In this regard, a BMI-related mediator effect was suggested, but carbohydrate intake did not show any relationship with BMI in the present study. Similar to the present study, a U-shaped association was reported in a recent nationwide cohort study in China [8]. In addition, a nonlinear relationship was also reported in a meta-analysis that included eight studies regarding total carbohydrates and type 2 diabetes. However, it was found that the risk of type 2 diabetes was lower at the extremes of carbohydrate intake (inverted U-shape). That study suggested that healthier and more active people are likely to consume more carbohydrates [26]. In the present study, participants with higher carbohydrate intake tended to be nonsmokers and nondrinkers and to have a high level of physical activity, as is consistent with the findings of other studies [8,10,12]. Therefore, these characteristics may have influenced the results. The Asian population is known to have a higher risk of diabetes than the European population at low BMI levels. It has been suggested that the consumption of refined carbohydrates such as white rice is associated with a ‘normal-weight metabolically obese’ phenotype in Asians, which may contribute to an increased risk of diabetes [27]. Moreover, in this study, BMI and WC were found to be independently related to incident diabetes (data not shown). Therefore, it is definitely necessary to reduce obesity to prevent diabetes.

The effectiveness of isocaloric macronutrient substitution is frequently evaluated in macronutrient studies because changes in one macronutrient are replaced by compensatory changes in another macronutrient if the total energy is fixed. In this study, that replacement of fats with carbohydrates was associated with an increased risk of diabetes. Contrary to our findings, the substitution of carbohydrates for fats or proteins was inversely associated with the risk of diabetes in a previous study [10]. In a European study, the replacement of 5 E% protein intake with isocaloric carbohydrates was associated with a 23% lower risk of diabetes; this lower risk was not observed when fats were replaced with carbohydrates [12]. In a study that used data from the EPIC study, in addition to its association with diabetes, the substitution of carbohydrates for fats showed a positive association with cardiovascular disease mortality in diabetic patients (HR 1.07, 95% CI: 1.02–1.13) [24].

The differences in observed associations among studies are presumably related to differences in mean carbohydrate intake. In general, the percentage of energy obtained from carbohydrates is lower in North American and European (mean <50 E%) countries than in Asian countries (mean >60 E%) [6]. However, carbohydrates are correlated with fat intake, such that lower mean carbohydrate intake is indicative of higher fat intake (E% fat: 21.0 E% in Korea in 2016–2017 [28], 34.8 E% in the US during 2013–2016 [29], and 28.5–46.2 E% in Europe [30]). The mean carbohydrate intake in the present study was 71.1 E%, which is higher than in a recent Asian study [8]. In addition, the health risks of replacing fats with carbohydrates differed according to fatty acid type. The substitution of carbohydrates for polyunsaturated fatty acids [12] or trans fatty acids [10] was generally associated with a decreased risk of diabetes, but not always. However, in the present study, the replacement of fatty acids with carbohydrates was not evaluated because of insufficient data. Thus, further research is needed. Additionally, to better understand the effects of macronutrients on the pathogenesis of diabetes, further studies are needed to determine the association of macronutrients substitution with plasma C-peptide [31], circulating glucagon levels [32], and amylase [16] involved in glucose metabolism.

Several factors should be considered when interpreting the results. First, the data do not represent the entire Korean population; therefore, the generalizability of the findings is limited. Although dietary data were collected using a validated FFQ, measurement errors could have affected the observed associations. Data collected through self-reporting may contain inaccuracies, which may have affected associations. Various covariates were considered, but there remain residual confounding effects caused by unmeasured covariates (e.g., supplements). Finally, the follow-up loss presumably occurred because of the cohort study design, and there was a possibility of unidentified bias. We did not suggest a specific interval for carbohydrate intake with a lower risk of diabetes. Further research using data representative of the Korean population is needed.

However, the study had several strengths. The findings were derived from a large-scale, long-term observational study. Carbohydrates were assessed using a continuous scale; they showed a U-shaped relationship with diabetes. There have been few studies concerning the effect of substituting macronutrients in the context of gene–macronutrient interactions [14]. In the present study, the modification effects of genetic variants on the association between replacing fats with carbohydrates and risk of diabetes were assessed; the relationships were not statistically significant.

## 5. Conclusions

In summary, this study found a nonlinear relationship between carbohydrate intake and incident diabetes, with the lowest risk observed at 78 E%. In addition, the risk of incident diabetes increased when fats were replaced with carbohydrates; however, this association did not depend on the 24 variants associated with type 2 diabetes.

## Figures and Tables

**Table 1:** Participant characteristics.

	Total	E% Quintiles of Carbohydrates			*p*-Value ^a^
		Q1	Q3	Q5	
*N*	7413	1483	1482	1482	
Sex					
Male	3507 (47.31%)	840 (56.64%)	738 (49.8%)	466 (31.44%)	<0.001
Female	3906 (52.69%)	643 (43.36%)	744 (50.2%)	1016 (68.56%)	
Age, years	51.53 (±8.72)	48.46 (±7.6)	50.85 (±8.28)	56.46 (±8.68)	<0.001
Rural region					
Yes	3621 (48.85%)	509 (34.32%)	617 (41.63%)	1189 (80.23%)	<0.001
No	3792 (51.15%)	974 (65.68%)	865 (58.37%)	293 (19.77%)	
Educational level					
Less than middle-school graduate	4025 (54.61%)	544 (36.83%)	782 (52.91%)	1181 (80.67%)	<0.001
Graduated high school	2334 (31.66%)	623 (42.18%)	493 (33.36%)	220 (15.03%)	
Some college or higher	1012 (13.73%)	310 (20.99%)	203 (13.73%)	63 (4.3%)	
Body mass index, kg/m^2^	24.42 (±3.09)	24.39 (±3.02)	24.37 (±3.03)	24.4 (±3.39)	0.774
≥25.0 kg/m^2^	3015 (40.67%)	593 (39.99%)	611 (41.23%)	600 (40.49%)	0.920
Waist circumference, cm	81.98 (±8.68)	81.4 (±8.6)	81.53 (±8.48)	83.16 (±9.15)	<0.001
≥90 cm for male and ≥85 cm for female	1966 (26.55%)	329 (22.23%)	359 (24.22%)	533 (35.96%)	<0.001
Current smoking status	1854 (25.21%)	452 (30.75%)	381 (25.81%)	264 (18.08%)	<0.001
Alcohol intake					
Nondrinker	3790 (52.36%)	562 (39.03%)	733 (50.21%)	1000 (69.83%)	<0.001
<15 g/day	2073 (28.64%)	456 (31.67%)	469 (32.12%)	306 (21.37%)	
15–24 g/day	500 (6.91%)	136 (9.44%)	93 (6.37%)	49 (3.42%)	
≥25 g/day	875 (12.09%)	286 (19.86%)	165 (11.3%)	77 (5.38%)	
Physical activity					
Q1	1648 (22.23%)	309 (20.84%)	320 (21.59%)	351 (23.68%)	<0.001
Q2	2013 (27.16%)	470 (31.69%)	428 (28.88%)	265 (17.88%)	
Q3	1906 (25.71%)	445 (30.01%)	409 (27.6%)	308 (20.78%)	
Q4	1846 (24.9%)	259 (17.46%)	325 (21.93%)	558 (37.65%)	
Total energy, kcal	1939.15 (±618.76)	2191.69 (±673.1)	1921.79 (±554.75)	1745.87 (±653.1)	<0.001
Carbohydrate, E%	71.08 (±6.96)	60.87 (±4.66)	71.57 (±1.03)	80.06 (±2.24)	<0.001
Protein, E%	13.43 (±2.33)	16.29 (±2.06)	13.28 (±1.2)	10.82 (±1.13)	<0.001
Fat, E%	14.41 (±5.4)	22.07 (±3.77)	14.07 (±1.59)	7.69 (±1.97)	<0.001
Carbohydrate, g ^b^	341.84 (±36.16)	291.95 (±29.91)	346.6 (±10.57)	380.51 (±26.64)	<0.001
Protein, g ^b^	65.72 (±11.92)	79.71 (±12.01)	64.28 (±6.5)	54.57 (±7.98)	<0.001
Fat, g ^b^	32.17 (±12.19)	48.4 (±10.26)	30.7 (±4.45)	19.55 (±9.26)	<0.001
Fiber, g/1000 kcal	3.61 (±1.23)	3.35 (±1.06)	3.62 (±1.18)	3.74 (±1.43)	<0.001

^a^*p*-values were calculated using the chi-squared test for categorical variables and Student’s *t*-test for numerical variables. ^b^ Energy-adjusted nutrient intake was estimated using the residual method.

**Table 2:** Risks of incident diabetes according to E% quintile of carbohydrates.

					Crude Model		Adjusted Model	
	Quintiles of Carbohydrates (E%)	PY	N	Cases (%)	HR (95% CI)	*p*-Value	HR (95% CI)	*p*-Value
Total	Q1	18160.19	1483	210 (14.16%)	1.00		1.00	
	Q2	18245.71	1483	242 (16.32%)	1.14 (0.95–1.37)	0.169	1.13 (0.94–1.36)	0.203
	Q3	18034.28	1482	240 (16.19%)	1.14 (0.95–1.37)	0.169	1.08 (0.89–1.31)	0.419
	Q4	18534.79	1483	245 (16.52%)	1.13 (0.94–1.35)	0.208	0.99 (0.81–1.20)	0.886
	Q5	18070.32	1482	256 (17.27%)	1.21 (1.01–1.45)	0.041	0.96 (0.78–1.19)	0.721
	trend				1.04 (1.00–1.08)	0.079	0.98 (0.93–1.03)	0.381
Male	Q1	10166.38	840	128 (15.24%)	1.00		1.00	
	Q2	9779.19	811	135 (16.65%)	1.09 (0.85–1.38)	0.511	1.09 (0.85–1.39)	0.512
	Q3	8770.00	738	128 (17.34%)	1.14 (0.89–1.46)	0.296	1.17 (0.91–1.50)	0.232
	Q4	7862.07	652	118 (18.10%)	1.17 (0.91–1.50)	0.217	1.17 (0.89–1.53)	0.255
	Q5	5553.52	466	82 (17.6%)	1.14 (0.86–1.50)	0.368	1.04 (0.76–1.43)	0.798
	trend				1.04 (0.98–1.10)	0.228	1.02 (0.96–1.10)	0.485
Female	Q1	7993.81	643	82 (12.75%)	1.00		1.00	
	Q2	8466.52	672	107 (15.92%)	1.23 (0.92–1.64)	0.156	1.22 (0.91–1.65)	0.181
	Q3	9264.28	744	112 (15.05%)	1.18 (0.89–1.57)	0.249	1.02 (0.76–1.37)	0.903
	Q4	10672.72	831	127 (15.28%)	1.15 (0.87–1.52)	0.312	0.86 (0.64–1.16)	0.325
	Q5	12516.80	1016	174 (17.13%)	1.37 (1.06–1.79)	0.018	0.90 (0.67–1.22)	0.502
	trend				1.06 (1.00–1.12)	0.049	0.94 (0.88–1.01)	0.090

HR, hazard ratio; 95% CI, 95% confidence interval; and PY, person-years. HRs with 95% CIs were estimated after adjustments for age, rural region, educational level, current smoking status, alcohol intake, physical activity, BMI, and total energy. The trend is the result of analysis of applying an ordinal independent variable as a continuous variable.

**Table 3:** Subgroup analysis for risk of incident diabetes when replacing fats with carbohydrates.

	Multivariate Nutrient Density Model (with Energy in the Model) ^a^		Nutrient Residual Model (with Energy in the Model) ^b^	
	HR (95% CI)	*p*-Value	HR (95% CI)	*p*-Value
Total fat	1.11 (1.01–1.21)	0.023	1.03 (1–1.07)	0.047
Sex				
Male	1.13 (1–1.28)	0.055	1.05 (1–1.09)	0.050
Female	1.09 (0.96–1.23)	0.165	1.03 (0.98–1.08)	0.247
Age				
< 50 years	1.05 (0.92–1.19)	0.480	1.01 (0.97–1.06)	0.598
≥ 50 years	1.16 (1.03–1.31)	0.014	1.06 (1.01–1.11)	0.017
Rural region				
Yes	1.09 (0.97–1.22)	0.146	1.02 (0.98–1.07)	0.242
No	1.11 (0.97–1.27)	0.128	1.04 (0.98–1.09)	0.171
Educational level				
Less than middle-school graduate	1.13 (1–1.26)	0.044	1.03 (0.99–1.08)	0.145
Graduated high school	0.99 (0.85–1.16)	0.927	1 (0.94–1.06)	0.970
Some college or higher	1.32 (1.03–1.68)	0.028	1.11 (1.02–1.21)	0.012
Body mass index, kg/m^2^				
<25.0 kg/m^2^	1.11 (0.97–1.26)	0.138	1.04 (0.99–1.1)	0.096
≥25.0 kg/m^2^	1.11 (0.99–1.25)	0.082	1.02 (0.98–1.07)	0.243
Abdominal obesity				
No	1.15 (1.03–1.29)	0.016	1.05 (1.01–1.1)	0.014
Yes	1.06 (0.92–1.21)	0.429	1.01 (0.96–1.06)	0.671
Current smoking status				
Yes	1.13 (0.96–1.32)	0.135	1.05 (0.99–1.11)	0.133
No	1.1 (0.99–1.23)	0.069	1.03 (0.99–1.07)	0.143
Physical activity				
<median	1.12 (0.98–1.28)	0.084	1.04 (0.99–1.1)	0.084
≥median	1.09 (0.97–1.23)	0.139	1.02 (0.98–1.07)	0.272
Fiber (g/1000 kcal)				
<median	1.07 (0.94–1.23)	0.307	1.03 (0.98–1.08)	0.236
≥median	1.05 (0.91–1.21)	0.514	1.00 (0.95–1.05)	0.951

HR, hazard ratio; 95% CI, 95% confidence interval. ^a^ HRs with 95% CIs were estimated after adjustments for sex, age, rural region, educational level, current smoking status, alcohol intake, physical activity, body mass index (BMI), total energy, and protein (per 5 E%). ^b^ HRs with 95% CIs were estimated after adjustments for age, rural region, educational level, current smoking status, alcohol intake, physical activity, BMI, total energy, and protein (per 10 g). Abdominal obesity was defined as a waist circumference of ≥90 cm for male and ≥85 cm for female.

## Data Availability

The KoGES data are available on request from the National Research Institute of Health [19].

## Supplementary Materials

The following supporting information can be downloaded at: https://www.mdpi.com/article/10.3390/nu14030654/s1, Table S1. Risks of incident diabetes for high carbohydrate intake (≥65 E% vs. <65 E%) according to genotype. Table S2. Risks of incident diabetes for high carbohydrate intake (≥65 E% vs. <65 E%) according to genotype and stratified according to sex. Table S3. Risks of incident diabetes when replacing fats with carbohydrates according to genotype and stratified according to energy adjusted model.
