# Supplementary material for: Substitution of Carbohydrates for Fats and Risk of Type 2 Diabetes among Korean Middle-Aged Adults: Findings from the Korean Genome and Epidemiology Study
Source: Nutrients. 2022 Feb 3;14(3):654. doi: 10.3390/nu14030654 (PMC8838696; doi:10.3390/nu14030654)
Supplement: Supplementary file 1 [file nutrients-14-00654-s001.zip › nutrients-1575041-supplementary.pdf]

Substitution of carbohydrates for fats and risk of type 2 diabetes among Korean middle-aged adults: findings from the Korean Genome and Epidemiology Study

Hye Ah Lee[1], Hyesook Park[2,3]

[1]Clinical Trial Center, Ewha Womans University Mokdong Hospital, Seoul, Republic of Korea,

[2]Department of Preventive Medicine, College of Medicine, Ewha Womans University, Seoul, Republic of Korea,

[3]Graduate Program in System Health Science and Engineering, Ewha Womans University, Seoul, Republic of Korea

Table S1. Risks of incident diabetes for high carbohydrate intake (≥ 65 E% *vs.* < 65 E%) according to genotype.

No	SNP	Genotype			*P*-value [a]	*P*-value [b]
		AA	Aa	aa		
		HR (95% CI)	HR (95% CI)	HR (95% CI)		
1	rs2233580	1.05 (0.87-1.28)	1.23 (0.80-1.87)	1.43 (0.60-3.37)	0.513	0.615
2	rs2206734	1.27 (0.93-1.73)	1.07 (0.90-1.29)	0.91 (0.68-1.23)	0.194	0.467
3	rs2237895	1.04 (0.81-1.33)	1.10 (0.90-1.35)	1.17 (0.79-1.75)	0.655	0.748
4	rs11960799	0.99 (0.76-1.28)	1.12 (0.92-1.36)	1.26 (0.87-1.84)	0.343	0.633
5	rs75680863	1.09 (0.90-1.32)	0.99 (0.58-1.70)	0.90 (0.31-2.67)	0.744	0.812
6	rs2237892	1.25 (0.96-1.63)	1.03 (0.85-1.24)	0.84 (0.59-1.22)	0.132	0.397
7	rs10965250	1.05 (0.80-1.37)	1.09 (0.91-1.32)	1.14 (0.80-1.62)	0.748	0.781
8	rs7901695	1.16 (0.96-1.41)	0.61 (0.37-0.99)	0.32 (0.12-0.85)	**0.014**	0.324
9	rs7754840	1.27 (0.93-1.75)	1.08 (0.90-1.30)	0.92 (0.69-1.24)	0.200	0.436
10	rs1470579	0.95 (0.75-1.20)	1.16 (0.94-1.43)	1.42 (0.94-2.14)	0.130	0.445
11	rs576674	1.00 (0.82-1.23)	1.30 (0.95-1.79)	1.70 (0.90-3.22)	0.142	0.378
12	rs7593730	1.22 (0.98-1.52)	0.88 (0.68-1.13)	0.63 (0.37-1.06)	**0.035**	0.278
13	rs5219	1.15 (0.88-1.52)	1.06 (0.88-1.27)	0.97 (0.69-1.36)	0.490	0.619
14	rs5015480	1.03 (0.83-1.28)	1.16 (0.90-1.51)	1.31 (0.78-2.20)	0.433	0.693
15	rs1387153	0.98 (0.74-1.29)	1.10 (0.91-1.32)	1.23 (0.87-1.75)	0.377	0.646
16	rs4430796	1.34 (1.03-1.74)	0.99 (0.81-1.19)	0.73 (0.50-1.05)	**0.021**	0.248
17	rs11634397	1.02 (0.84-1.23)	1.49 (0.96-2.31)	2.18 (0.90-5.30)	0.109	0.435
18	rs2943641	1.03 (0.85-1.24)	1.73 (0.94-3.20)	2.92 (0.85-10.04)	0.108	0.519
19	rs635634	1.00 (0.79-1.26)	1.16 (0.92-1.45)	1.34 (0.85-2.12)	0.310	0.621
20	rs4402960	0.92 (0.73-1.17)	1.19 (0.96-1.48)	1.54 (1.00-2.36)	0.062	0.370
21	rs10758593	1.16 (0.89-1.52)	1.06 (0.88-1.28)	0.97 (0.69-1.37)	0.477	0.673
22	rs1153188	1.08 (0.90-1.30)	1.10 (0.51-2.38)	1.12 (0.24-5.29)	0.964	0.964
23	rs757110	1.17 (0.88-1.54)	1.07 (0.89-1.28)	0.98 (0.70-1.36)	0.482	0.642
24	rs7177055	1.16 (0.89-1.52)	1.06 (0.88-1.28)	0.97 (0.68-1.37)	0.476	0.714

AA, major-allele homozygotes; Aa, heterozygotes, aa, minor-allele homozygotes
SNP, single nucleotide polymorphism; HR, hazard ratio; 95% CI, 95% confidence interval
[a] *P*-value for interaction between carbohydrate intake and genotypes
[b] Adjusted *P*-value calculated using the Benjamini–Hochberg method
HRs with 95% CIs were estimated after adjustments for age, rural region, educational level, current smoking status, alcohol intake, physical activity, BMI, and total energy.

Table S2. Risks of incident diabetes for high carbohydrate intake (≥ 65 E% *vs*. < 65 E%) according to genotype and stratified according to sex

No	SNP	Male					Female				
		AA	Aa	aa	*P*	*P*	AA	Aa	aa	*P*	*P*
		HR (95% CI)	HR (95% CI)	HR (95% CI)	value [a]	value [b]	HR (95% CI)	HR (95% CI)	HR (95% CI)	value [a]	value [b]
1	rs2233580	1.11 (0.87-1.43)	1.47 (0.85-2.55)	1.95 (0.64-5.92)	0.347	0.490	0.99 (0.73-1.35)	0.99 (0.51-1.94)	0.99 (0.26-3.85)	0.999	0.999
2	rs2206734	1.41 (0.95-2.10)	1.16 (0.92-1.46)	0.96 (0.64-1.42)	0.240	0.443	1.13 (0.69-1.87)	0.98 (0.74-1.31)	0.86 (0.54-1.36)	0.481	1.050
3	rs2237895	1.14 (0.83-1.57)	1.18 (0.91-1.54)	1.23 (0.73-2.07)	0.837	0.914	0.92 (0.62-1.36)	1.04 (0.75-1.44)	1.18 (0.63-2.23)	0.555	0.833
4	rs11960799	1.08 (0.78-1.50)	1.21 (0.94-1.55)	1.35 (0.82-2.22)	0.519	0.623	0.92 (0.61-1.39)	1.02 (0.75-1.39)	1.14 (0.64-2.01)	0.599	0.845
5	rs75680863	1.24 (0.97-1.59)	0.66 (0.34-1.29)	0.35 (0.09-1.35)	0.079	0.472	0.93 (0.69-1.25)	1.82 (0.66-5.01)	3.56 (0.46-27.50)	0.211	1.264
6	rs2237892	1.43 (1.02-2.01)	1.09 (0.85-1.39)	0.83 (0.52-1.32)	0.097	0.389	1.11 (0.72-1.71)	0.95 (0.70-1.29)	0.82 (0.45-1.48)	0.482	0.963
7	rs10965250	1.17 (0.83-1.66)	1.17 (0.92-1.49)	1.17 (0.75-1.83)	0.993	0.993	0.89 (0.58-1.36)	1.02 (0.75-1.39)	1.17 (0.65-2.11)	0.514	0.948
8	rs7901695	1.25 (0.97-1.59)	0.70 (0.37-1.36)	0.40 (0.11-1.51)	0.108	0.372	1.09 (0.80-1.48)	0.49 (0.24-1.00)	0.22 (0.05-0.94)	**0.041**	0.490
9	rs7754840	1.33 (0.90-1.97)	1.16 (0.92-1.47)	1.02 (0.69-1.52)	0.424	0.565	1.30 (0.76-2.22)	1.01 (0.75-1.36)	0.79 (0.51-1.22)	0.213	1.021
10	rs1470579	0.99 (0.73-1.35)	1.28 (0.98-1.68)	1.65 (0.96-2.82)	0.149	0.324	0.90 (0.62-1.31)	1.05 (0.75-1.45)	1.22 (0.64-2.30)	0.470	1.128
11	rs576674	1.03 (0.79-1.33)	1.64 (1.06-2.53)	2.62 (1.08-6.33)	0.057	0.459	0.99 (0.71-1.37)	1.00 (0.63-1.58)	1.00 (0.40-2.51)	0.981	1.024
12	rs7593730	1.36 (1.02-1.81)	0.92 (0.67-1.27)	0.62 (0.32-1.20)	0.050	0.604	1.07 (0.76-1.51)	0.86 (0.55-1.34)	0.69 (0.27-1.73)	0.420	1.260
13	rs5219	1.46 (1.01-2.09)	1.10 (0.87-1.40)	0.84 (0.55-1.29)	0.090	0.431	0.87 (0.57-1.34)	1.03 (0.76-1.39)	1.21 (0.68-2.17)	0.430	1.147
14	rs5015480	1.06 (0.80-1.39)	1.37 (0.98-1.91)	1.78 (0.91-3.45)	0.186	0.371	1.03 (0.73-1.44)	0.93 (0.62-1.41)	0.85 (0.37-1.95)	0.702	0.886
15	rs1387153	1.04 (0.73-1.50)	1.19 (0.94-1.51)	1.37 (0.87-2.13)	0.424	0.536	0.91 (0.59-1.40)	1.01 (0.75-1.36)	1.13 (0.64-2.00)	0.605	0.806
16	rs4430796	1.63 (1.17-2.26)	0.97 (0.75-1.24)	0.57 (0.35-0.94)	**0.002**	0.055	1.03 (0.67-1.60)	1.00 (0.74-1.35)	0.97 (0.54-1.73)	0.884	1.061
17	rs11634397	1.11 (0.87-1.42)	1.53 (0.88-2.68)	2.12 (0.69-6.52)	0.281	0.450	0.92 (0.68-1.25)	1.50 (0.72-3.11)	2.44 (0.56-10.64)	0.216	0.865

18	rs2943641	1.10 (0.87-1.39)	2.22 (0.95-5.19)	4.47 (0.81-24.79)	0.117	0.313	0.96 (0.72-1.30)	1.28 (0.53-3.08)	1.71 (0.29-9.94)	0.538	0.922
19	rs635634	1.06 (0.79-1.42)	1.29 (0.95-1.73)	1.56 (0.85-2.87)	0.303	0.454	0.98 (0.67-1.44)	1.00 (0.71-1.42)	1.03 (0.51-2.07)	0.924	0.999
20	rs4402960	1.00 (0.74-1.35)	1.29 (0.98-1.71)	1.68 (0.96-2.94)	0.144	0.347	0.85 (0.59-1.23)	1.10 (0.78-1.55)	1.42 (0.73-2.77)	0.232	0.796
21	rs10758593	1.01 (0.73-1.41)	1.23 (0.96-1.57)	1.48 (0.93-2.37)	0.249	0.427	1.50 (0.94-2.39)	0.92 (0.69-1.23)	0.57 (0.35-0.94)	**0.015**	0.350
22	rs1153188	1.17 (0.92-1.48)	1.15 (0.39-3.40)	1.13 (0.13-9.96)	0.977	1.019	0.99 (0.74-1.33)	1.06 (0.35-3.22)	1.13 (0.12-10.63)	0.910	0.999
23	rs757110	1.46 (1.01-2.12)	1.14 (0.90-1.44)	0.88 (0.59-1.33)	0.116	0.348	0.90 (0.58-1.37)	1.02 (0.75-1.37)	1.15 (0.65-2.04)	0.541	0.865
24	rs7177055	1.14 (0.81-1.59)	1.18 (0.92-1.50)	1.22 (0.77-1.93)	0.835	0.954	1.34 (0.84-2.12)	0.92 (0.69-1.24)	0.64 (0.38-1.09)	0.074	0.594

AA, major-allele homozygotes; Aa, heterozygotes, aa, minor-allele homozygotes

SNP, single nucleotide polymorphism; HR, hazard ratio; 95% CI, 95% confidence interval

[a] *P*-value for interaction between carbohydrate intake and genotypes

[b] Adjusted *P*-value calculated using the Benjamini–Hochberg method

HRs with 95% CIs were estimated after adjustments for age, rural region, educational level, current smoking status, alcohol intake, physical activity, BMI, and total energy.

Table S3. Risks of incident diabetes when replacing fats with carbohydrates according to genotype and stratified according to energy adjusted model

No	SNP	Multivariate nutrient density model (with energy in the model) [c]					Nutrient residual model (with energy in the model) [d]				
		AA	Aa	aa	*P* value [a]	*P* value [b]	AA	Aa	aa	*P* value [a]	*P* value [b]
		HR (95% CI)	HR (95% CI)	HR (95% CI)			HR (95% CI)	HR (95% CI)	HR (95% CI)		
1	rs2233580	1.12 (1.02-1.24)	1.18 (1.03-1.34)	1.24 (0.99-1.54)	0.389	0.934	1.04 (1.00-1.07)	1.05 (0.99-1.10)	1.06 (0.97-1.15)	0.699	0.999
2	rs2206734	1.15 (1.03-1.29)	1.13 (1.03-1.24)	1.11 (0.99-1.24)	0.555	0.784	1.05 (1.00-1.09)	1.04 (1.00-1.07)	1.03 (0.99-1.07)	0.470	0.999
3	rs2237895	1.10 (0.99-1.23)	1.14 (1.03-1.26)	1.18 (1.04-1.35)	0.310	0.999	1.03 (0.99-1.07)	1.04 (1.00-1.08)	1.05 (0.99-1.10)	0.631	0.999
4	rs11960799	1.11 (1.00-1.24)	1.14 (1.03-1.25)	1.16 (1.02-1.32)	0.536	0.804	1.03 (0.99-1.07)	1.04 (1.01-1.08)	1.06 (1.01-1.11)	0.193	0.999
5	rs75680863	1.13 (1.03-1.25)	1.09 (0.93-1.28)	1.05 (0.79-1.40)	0.621	0.784	1.04 (1.00-1.07)	1.03 (0.97-1.10)	1.03 (0.92-1.15)	0.877	0.999
6	rs2237892	1.13 (1.02-1.26)	1.13 (1.02-1.24)	1.12 (0.99-1.28)	0.877	0.877	1.04 (1.00-1.08)	1.04 (1.00-1.07)	1.03 (0.98-1.08)	0.807	0.999
7	rs10965250	1.11 (1.00-1.23)	1.14 (1.04-1.26)	1.18 (1.04-1.33)	0.373	0.995	1.04 (1.00-1.08)	1.04 (1.00-1.08)	1.04 (0.99-1.09)	0.957	0.957
8	rs7901695	1.15 (1.04-1.26)	0.98 (0.81-1.18)	0.84 (0.59-1.18)	0.076	0.999	1.05 (1.01-1.08)	0.98 (0.92-1.05)	0.92 (0.82-1.04)	0.058	0.999
9	rs7754840	1.14 (1.02-1.28)	1.13 (1.03-1.24)	1.11 (0.99-1.25)	0.681	0.743	1.05 (1.00-1.09)	1.04 (1.00-1.07)	1.03 (0.99-1.07)	0.437	0.999
10	rs1470579	1.11 (1.00-1.23)	1.14 (1.03-1.26)	1.17 (1.02-1.34)	0.431	0.796	1.03 (0.99-1.08)	1.04 (1.00-1.07)	1.04 (0.99-1.09)	0.825	0.999
11	rs576674	1.11 (1.01-1.23)	1.17 (1.04-1.31)	1.23 (1.03-1.47)	0.272	0.999	1.03 (0.99-1.07)	1.05 (1.01-1.09)	1.07 (1.00-1.14)	0.310	0.999
12	rs7593730	1.16 (1.05-1.28)	1.08 (0.97-1.20)	1.00 (0.85-1.18)	0.087	0.999	1.05 (1.01-1.08)	1.02 (0.98-1.06)	0.99 (0.93-1.06)	0.121	0.999
13	rs5219	1.15 (1.03-1.28)	1.12 (1.02-1.24)	1.10 (0.98-1.24)	0.480	0.823	1.04 (1.00-1.08)	1.04 (1.00-1.07)	1.04 (0.99-1.08)	0.898	0.980
14	rs5015480	1.11 (1.00-1.23)	1.16 (1.04-1.29)	1.22 (1.04-1.42)	0.226	0.999	1.03 (0.99-1.07)	1.05 (1.00-1.09)	1.06 (1.00-1.13)	0.390	0.999
15	rs1387153	1.11 (0.99-1.24)	1.13 (1.03-1.25)	1.15 (1.02-1.30)	0.590	0.787	1.03 (0.99-1.07)	1.04 (1.00-1.08)	1.05 (1.00-1.10)	0.410	0.999
16	rs4430796	1.14 (1.03-1.27)	1.12 (1.02-1.24)	1.10 (0.96-1.26)	0.628	0.754	1.04 (1.00-1.08)	1.03 (1.00-1.07)	1.03 (0.97-1.08)	0.594	0.999

17	rs11634397	1.12 (1.02-1.23)	1.18 (1.04-1.35)	1.25 (1.00-1.57)	0.304	0.999	1.03 (1.00-1.07)	1.06 (1.01-1.11)	1.08 (0.99-1.18)	0.285	0.999
18	rs2943641	1.12 (1.02-1.23)	1.20 (1.03-1.40)	1.28 (0.97-1.69)	0.324	0.972	1.03 (1.00-1.07)	1.06 (1.00-1.12)	1.09 (0.97-1.21)	0.374	0.999
19	rs635634	1.11 (1.00-1.23)	1.15 (1.04-1.27)	1.18 (1.03-1.36)	0.390	0.851	1.03 (1.00-1.07)	1.04 (1.00-1.08)	1.05 (0.99-1.10)	0.632	0.999
20	rs4402960	1.09 (0.98-1.21)	1.15 (1.04-1.27)	1.21 (1.05-1.38)	0.160	0.999	1.03 (0.99-1.07)	1.04 (1.00-1.08)	1.06 (1.00-1.11)	0.287	0.999
21	rs10758593	1.12 (1.01-1.25)	1.13 (1.03-1.25)	1.14 (1.01-1.30)	0.758	0.791	1.03 (0.99-1.08)	1.04 (1.00-1.08)	1.04 (1.00-1.09)	0.685	0.999
22	rs1153188	1.13 (1.02-1.24)	1.22 (0.96-1.55)	1.32 (0.84-2.09)	0.489	0.782	1.04 (1.00-1.07)	1.03 (0.95-1.13)	1.03 (0.87-1.22)	0.933	0.974
23	rs757110	1.16 (1.04-1.30)	1.13 (1.03-1.24)	1.10 (0.98-1.24)	0.397	0.794	1.04 (1.00-1.09)	1.04 (1.00-1.08)	1.04 (0.99-1.08)	0.753	0.999
24	rs7177055	1.12 (1.00-1.24)	1.13 (1.03-1.25)	1.15 (1.02-1.31)	0.631	0.721	1.04 (0.99-1.08)	1.04 (1.00-1.07)	1.04 (0.99-1.09)	0.844	0.999

AA, major-allele homozygotes; Aa, heterozygotes, aa, minor-allele homozygotes

SNP, single nucleotide polymorphism; HR, hazard ratio; 95% CI, 95% confidence interval

[a] *P*-value for interaction between carbohydrate intake and genotypes

[b] Adjusted *P*-value calculated using the Benjamini–Hochberg method

[c] HRs with 95% CIs were estimated after adjustments for sex, age, rural region, educational level, current smoking status, alcohol intake, physical activity, body mass index (BMI), total energy, and protein (per 5 E%).

[d] HRs with 95% CIs were estimated after adjustments for age, rural region, educational level, current smoking status, alcohol intake, physical activity, BMI, total energy, and protein (per 10 g).
